# Development and validation of the conversation questionnaire: A psychometric measure of communication challenges generated from the self-reports of autistic people

**DOI:** 10.1177/23969415221123286

**Published:** 2022-09-04

**Authors:** Alexander C Wilson

**Affiliations:** Department of Experimental Psychology, 6396 University of Oxford, Oxford, UK; School of Psychology, Newcastle University, Newcastle Upon Tyne, UK

**Keywords:** Autism, social communication, conversation, pragmatics, psychometrics, community engagement

## Abstract

Existing measures of communication challenges in autism are based on diagnostic criteria and research/clinical observations of autistic people, rather than what autistic people themselves identify as difficulties. In this study, the Conversation Questionnaire (CQ) was developed based on community engagement with autistic people to identify what they find challenging about conversation. This new tool was then administered online to autistic, dyslexic and neurotypical people (*N* = 312) in a validation phase of the study. Item-response theory modelling indicated that a two-dimensional structure accounted for response patterns. These dimensions reflected difficulties knowing what to say (15 items) and engaging in behaviours possibly disruptive to neurotypical conversation (21 items). The dimensions showed good internal consistency and convergent and discriminant validity, and could distinguish between autistic and neurotypical people (*d* = 1.59 and *d* = 2.07 respectively). The CQ might help contribute to diagnostic assessment for autism in adults as part of a holistic assessment. The questionnaire might also be useful with other neurodiverse groups, and provide a tool for clinicians and researchers to identify individuals’ strengths and difficulties in conversation (e.g., as part of interventions in speech and language therapy).

For a diagnosis of autism, individuals will show some differences in social communication compared to their peers (American Psychiatric Association, 2013). These differences may be most evident in conversation with neurotypical people, which can be a particular area of stress and challenge according to autistic people (Cummins et al., 2020; Kelly et al., 2018). Various features of conversation have been linked to autism. For example, individuals might assume knowledge in the conversation partner, provide considerable detail, shift the topic abruptly, make conversational turns that are not clearly linked to previous turns, dominate the conversation, struggle to initiate and maintain a conversation, and become preoccupied with particular topics (e.g., Bauminger-Zviely et al., 2014; Eales, 1993; Jones & Schwartz, 2009; Klusek et al., 2014; Lam & Yeung, 2012; Paul et al., 2009). Together these behaviours relate to social/pragmatic aspects of conversation (“pragmatic” typically refers to the process of communicating intended meanings in the conversational context; Wilson & Sperber, 2004). This paper reports on development of a novel measure of pragmatic/social aspects of conversation: the Conversation Questionnaire (CQ). Unlike other tools, this self-report questionnaire was devised through community engagement with autistic people, and focuses on features of conversation autistic people themselves report as challenging. The hope is that this questionnaire might help contribute to diagnostic assessment for autism in adults as part of a holistic assessment, as well as provide a tool for clinicians and researchers to identify individuals’ strengths and difficulties in conversation (e.g., as part of interventions in speech and language therapy). It is also worth noting that pragmatics/social communication can be an area of difference for other neurodiverse groups (such as people with language disorders or attention deficit hyperactivity disorder (ADHD); Geurts et al., 2004; Leyfer et al., 2008), and the tool might have use with these individuals too.

The first potential use of the CQ would be in supporting an assessment for autism. When assessing for autism in adults, clinicians rely on a combination of self/informant report questionnaires (e.g., Autism-Spectrum Quotient; Baron-Cohen et al., 2001) and diagnostic measures. Diagnostic measures include (1) structured observations of the individual (typically using the Autism Diagnostic Observation Schedule, Second Edition (ADOS-2); Lord et al., 2012) and (2) interviews about the individual’s developmental history and current presentation (e.g., Autism Diagnostic Interview, Revised (ADI-R); Rutter et al., 2003). Guidelines by the National Institute for Health and Care Excellence (NICE, 2012) advise that a battery of measures are used in assessing for autism (rather than relying on a single test), as measures rarely show strong specificity for autism, especially in clinically-referred samples, and there is a lack of research into the psychometric properties of measures (Wigham et al., 2019). There are further complications when assessing adults. These include the fact that early developmental information may not always be available, and the individual’s presentation may be more subtle than in autistic adults diagnosed as children (Lai & Baron-Cohen, 2015). Therefore, when assessing for autism in adults, it is critical to have multiple well-validated measures sensitive to subtle current features of autism. The CQ was devised to capture subtle current difficulties, as experienced and reported by autistic people, so it may offer a useful addition to a holistic assessment for autism.

The second potential use of the CQ would be assessing and helping individuals understand their profile of conversation skills, needs and preferences. Existing measures of conversation skills include self/informant report questionnaires (e.g., Communication Checklist – Self Report (CC-SR); Bishop et al., 2009), structured observations in response to communication probes (e.g., Yale in vivo Pragmatic Protocol; Schoen Simmons et al., 2014), rating scales used with semi-structured observations (e.g., Pragmatic Rating Scale; Landa et al., 1992) and formal language tests targeting pragmatics (e.g., Test of Pragmatic Language; Phelps-Terasaki & Phelps-Gunn, 2007). A significant limitation of all these measures (except the CC-SR) is that they lack norms and psychometric information for adults, and/or are only appropriate for use with children. In addition, there are questions relating to the validity of assessing pragmatics with observational measures and formal language tests (Adams, 2002; Norbury, 2014). Observational measures only provide a snapshot of an individual’s skills in a specific context, typically with a single sympathetic communication partner, and may therefore not represent the range of interactions with which the individual might struggle. Meanwhile, formal language tests may not be able to disentangle pragmatics from other aspects of language function and may be too structured to capture the context-dependent nature of pragmatics, and therefore their validity has been questioned (Adams, 2002; Norbury, 2014). Questionnaires may have the advantage over other types of measures of giving a meaningful summary of difficulties an individual experiences in everyday life (but they are, of course, subject to other limitations, such as reporting biases). Of all existing questionnaires, the CC-SR most specifically targets conversation skills, whereas other measures (e.g., Social Responsiveness Scale, Second Edition; Constantino, 2012) assess communication alongside a broader assessment of social interaction. However, use of the CC-SR in previous research has indicated some limitations: in one study, some autistic participants informally commented that the CC-SR seemed to classify difficulties from a non-autistic perspective, and participants often felt they needed to leave items as missing as they were difficult to judge (Wilson & Bishop, 2020). Devising a questionnaire with autistic people may help us overcome these issues.

So far, we have reviewed some of the limitations of measures used for (a) identifying autism, and (b) measuring conversational skills/social communication. NICE (2012) recommends the use of multiple types of measures when assessing for autism, partly due to the limitations intrinsic to any one form of assessment; and as highlighted, there seems to be a particular lack of appropriate measures for assessing conversational skills in adults. There is therefore room for a new measure of conversational skills in adults, and with this in mind, the self-report CQ was developed based on direct input of autistic people about what they find challenging about conversation. Of course, this is not meant to imply that the CQ is without limitations. Self-report measures like the CQ will always be subject to biases due to a person’s level of insight and perception of themselves (which may, for instance, be overly critical). As such, the CQ is best viewed as a tool that may provide additional information to a holistic assessment for autism. In addition, it is worth noting that the CQ may be useful with other neurodiverse groups who also experience challenges with social communication. The CQ was devised with autistic people, but this study did present the measure to dyslexic individuals as well to explore the extent to which responses on the CQ were specific to autistic people or overlapped with other neurodiverse people. Therefore, we should not ignore the possible relevance of the CQ for other groups too.

In considering the nature of the CQ, there are two issues to touch on here: why base the measure on the input of autistic people, and why use self-report? Existing measures have been devised based on diagnostic criteria, clinical observations, and the wider research literature rather than direct input from autistic people. From the perspective of participatory research, it is critical to build materials based on the lived experience of autistic people so that materials are collaborative, inclusive and relevant to the autistic community (Chown et al., 2017). It is likely to give a fuller understanding of a construct if we explore how it feels from the inside – for instance, our understanding of “masking” (where an individual uses learned strategies to blend in social situations) depends very much on autistic self-report (Hull et al., 2017b). In listening to the ways autistic people express their difficulties, it is also likely to make the measure as accessible as possible (Nicolaidis et al., 2015).

As for the second question, “why self-report”, self-report questionnaires offer important, often reliable information to an autism assessment, while also respecting the individual’s capacity to self-reflect. Self and informant report have shown reasonable convergence in adults (around 0.50; Horwitz et al., 2016; Sandercock et al., 2020) and combining scores from self and informant sources more strongly predicts psychosocial outcomes than relying on one reporter (Sandercock et al., 2020). In addition, if there are discrepancies between self and informant report, this may give meaningful information (e.g., about insight). Autistic people may also have a different perspective to offer about their experiences of conversation compared to (neurotypical) informants. In this respect, autistic people may perceive the social/pragmatic “problems” linked to autism as less significant than neurotypical people do (Sng et al., 2020). This is likely to impact communication between autistic and neurotypical people, so it might be important to understand how people perceive the conversations they have. A self-report tool such as the CQ could be a useful starting point for clinicians in discussing these issues with individuals.

This study reports creation and initial validation of the CQ. In *Part I* of the study, questionnaire items were developed based on the self-report of autistic adults, or directly suggested by autistic adults, and then were reviewed in partnership with autistic people in a survey. *Part II* was a validation phase, where the questionnaire was presented in an online survey to a large sample of people, including individuals with an autism diagnosis. Alongside the questionnaire, individuals completed some language tasks and other questionnaires. Validation of the CQ was a secondary purpose of this survey. The main purpose was to compare performance of autistic, dyslexic and neurotypical people on the language tasks for a registered report (Wilson & Bishop, 2022). However, a range of measures were included in *Part II* so that we could explore the validity of the CQ, including whether it showed a relationship with theoretically linked variables (e.g., autistic traits) but less relationship with theoretically distinct variables (e.g., core language/literacy difficulties). It was proposed that the CQ would show good face validity and acceptability when presented to autistic people in *Part I* of the study. In *Part II*, it was hypothesised that the CQ would show high reliability, high sensitivity and specificity for differentiating autistic and neurotypical people, and would show convergent and discriminant validity when assessed against other measures.

## Part I: Questionnaire development

### Method

This stage of the project received ethical approval from the Medical Sciences Division of the Oxford University Research Ethics Committee in November 2018.

### Participants

Sixty-five autistic adults were recruited for a study about language and communication (Wilson & Bishop, 2020) through support and social groups, and through Autistica, a research-focused charity in the UK. Inclusion criteria for individuals giving informed consent to participate included: (i) an autism spectrum diagnosis by a clinical service, (ii) native-level fluency in English, (iii) age of 18 years or over, (iv) no significant visual or hearing impairment, and (v) no history of neurological illness or head injury. Individuals were invited to participate regardless of other diagnoses, including ADHD, genetic syndromes or learning disabilities. The study opened in 2019 and all individuals expressing interest in participating by 31st March 2019 were invited to do so. When participants met with the researcher, they were asked details about their autism diagnosis, including how, where, when and by whom it was made. Each person reported a clinical diagnosis by appropriately trained professionals (clinical psychologists, psychiatrists and specialist nurse practitioners trained in autism diagnosis) and mostly as part of multidisciplinary teams (MDTs) in National Health Service (NHS) settings.

Forty-one individuals identified as female, 23 as male and one as non-binary. Average age was 39 years (*SD* = 14 years, min = 18 years, max = 70 years). The approximate average age at diagnosis was 31 years (*SD* = 18 years, 45 individuals were diagnosed as adults). Except for one Asian person, each autistic participant was White. The highest level of education was given as at least some high school/secondary school for 10 individuals; 6 individuals had or were completing vocational qualifications; 10 individuals indicated they had completed their education with some college/undergraduate education; 22 individuals had or were completing an undergraduate degree; and 16 individuals had a higher degree (one person did not answer this question).

### Procedure

Participants took part in an interview-based assessment using Module 4 of the Autism Diagnostic Observation Schedule (ADOS-2; Lord et al., 2012). This was part of a broader study on language and communication, and participants were briefed that the research focus was their communication experiences. The ADOS-2 was administered by a researcher trained to clinical and research level reliability on the ADOS-2, and assessments were recorded by video. The ADOS-2 includes some questions about social difficulties, and many participants disclosed challenges with communication and conversation in response to these. If this happened, participants were encouraged to elaborate further on their insights through open questions. Following administration of the ADOS-2, participants were asked if they had noticed themselves having difficulties with conversation (generally or in the course of the assessment). This gave them a further open opportunity to disclose experiences of conversation.

When watching back the videos, the researcher noted down verbatim conversation challenges described by each participant. These were then formatted into questionnaire items for the CQ. The aim was to keep closely to participants’ wording, though some items were shortened or simplified for readability, and repetition was avoided across items. When devising the questionnaire, it was kept in mind that respondents may sometimes respond to items indiscriminately, e.g., endorsing every single item. If this happens, it is difficult to know if individuals are genuinely reporting problems or, for instance, are just answering without processing the questions fully. Therefore, a few control items measuring Core Language Difficulties and Negative Interaction Style were included in the CQ. Individuals with social-pragmatic difficulties might not necessarily be expected to endorse these items, as difficulties with pragmatic aspects of communication are dissociable from language impairments (Whitehouse et al., 2007) and psychopathic traits (Rogers et al., 2006).

After putting together the questionnaire, participants were contacted to see what they thought about it. This was approximately six months after participants had taken part in the ADOS-2 assessments, and participation in this follow-up was entirely optional. If individuals were interested, they were invited to complete the CQ and then provide some feedback about it. For the feedback, participants were asked (1) if they understood the items, (2) if the answer format was easy to use, (3) if the length was appropriate, (4) if items felt relevant to them, (5) if there were important conversation difficulties not included in the questionnaire, (6) if the wording was respectful and sensitive, and (7) if they had any further feedback.

### Results

Of the 65 people who took part in an ADOS-2 assessment, 21 people (32%) agreed to participate anonymously in the optional follow-up where they were asked to give feedback about the newly-devised CQ. All participants responded positively to feedback questions (1), (2), (3) and (6); i.e., everyone reported understanding the items and the response format, and felt the length was appropriate and the wording respectful. In response to feedback question (4), which asked whether the questionnaire felt relevant to them, 14 people said “yes”; three said “mostly”; three said “some(times)” and one said “partly”. Based on suggestions made by participants in response to feedback question (5), 12 further items were added to the questionnaire, since these were conversational difficulties participants felt had not been covered in the questionnaire. As participants thought the original questionnaire was a good length, this length was maintained, so 11 items were removed to make room for the new ones. Items were removed on the basis that participants reported they were ambiguous (three items), or because item correlations indicated that particular items were redundant (five items) or entirely unrelated to other items (three items). The final version of the questionnaire was circulated among participants who gave feedback.

## Part II: Questionnaire validation

### Method

This stage of the project received ethical approval from the Medical Sciences Division of the Oxford University Research Ethics Committee in March 2020.

#### Participants

Autistic and non-autistic individuals were recruited according to the following eligibility criteria: (i) age of 18 years or over, (ii) native-level fluency in English, (iii) no history of acquired brain injury, (iv) no significant uncorrected sensory impairment, and (v) access to a computer with internet and audio. Three hundred and twenty people were recruited. Participants’ responses were retained in the dataset if they answered at least 90% of the social/pragmatic items on the CQ, which resulted in a sample of 312 people (i.e. eight people were excluded at this stage due to incomplete responses). One hundred and eighty four participants identified as female; 118 as male; and 10 as non-binary. Average age was 39 years (*SD* = 15 years, min = 18 years, max = 79 years). Two hundred and forty participants indicated they were White; 19 as Mixed Race; 14 as Asian; 10 as Black; and 29 people did not indicate their race (for instance, just indicating they were British). One hundred and ninety-one individuals (61%) reported that they had completed a Bachelor’s degree, and an additional 30 (10%) people indicated that they had completed secondary education and were currently undergraduate students. Participants were recruited between February and November 2021.

Participants were split into five groups. The first group (*N* = 101) included individuals reporting a diagnosis of autism. As part of the survey, participants were asked how, where, when and by whom their diagnosis was made. All diagnoses were made in a clinical service by a multidisciplinary team or an appropriately trained individual, such as a clinical psychologist, psychiatrist or developmental paediatrician. The second group (*N* = 34) included individuals self-identifying as autistic but who had no formal diagnosis. Formally and self-diagnosed individuals were grouped separately in case there were meaningful differences. It was felt to be important to include self-diagnosed people in the study, as this is a group that commonly experiences exclusion (Lewis, 2016). In addition to autistic people, a neurodiverse control group was recruited (the third group). This group included individuals with reading difficulties/dyslexia. As questionnaires currently used with autistic people often show low specificity when clinical control groups are used as the comparison condition (Wigham et al., 2019), it seemed useful to identify how well the CQ differentiated between autistic people and another group of neurodiverse people. As the core difficulties in dyslexia (i.e., with language/literacy) may be somewhat dissociable from the social/pragmatic challenges of autistic people, dyslexic individuals were felt to be an appropriate comparison group. However, it was held in mind that neurodevelopmental conditions show overlapping features, are heterogeneous, and people often have more than one neurodevelopmental diagnosis (Thapar et al., 2017). Therefore, it was possible that the dyslexic group would endorse features on the CQ, so the questionnaire might also show utility in assessing communication among individuals with other forms of neurodiversity such as dyslexia. For inclusion in this third group (*N* = 49), individuals needed to score above threshold on the reading scale of the Adult Reading Questionnaire (ARQ) but below threshold on the ten-item version of the Autism-Spectrum Quotient (AQ-10), which was 6 on each questionnaire). A fourth group (*N* = 110) included individuals without any neurodevelopmental diagnosis and below-threshold scores on the AQ-10 and ARQ. The fifth group (*N* = 18) included any non-autistic people excluded from the fourth or fifth groups; i.e., people in this group had elevated autistic traits, as reflected in an above-threshold AQ-10 score (but did not have an autism diagnosis or identify as autistic). This fifth group was retained in the study so that participants were not arbitrarily excluded, which might introduce bias into the results, while also reducing the likelihood that individuals with unidentified autism were present in the third and fourth groups. Demographic information for these five groups is shown in Table 1.

**Table 1.** Demographic information for each group.

	Group 1, Autistic (*N* = 101)		Group 2, Self-diagnosed autistic (*N* = 34)		Group 3, Reading difficulties (*N* = 49)		Group 4, Control (*N* = 110)		Group 5, Non-autistic high AQ (*N* = 18)	
Age in years (Mean, *SD*)	41.69	14.28	41.94	13.31	34.61	15.51	38.11	14.45	39.67	13.58
Females (*N*, %)	49.5	50	14	41	33	67	80	73	7	39
Males (*N*, %)	44.5	45	17	50	15	31	30	27	11	61
Non-binary (*N*, %)	6	6	3	9	1	2	0	0	0	0

Autistic individuals were recruited through Autistica, the research network for families and individuals with autism. Individuals with reading difficulties were recruited through charitable organisations such as the Helen Arkell Centre and Dyslexia Scotland, as well as social media. Non-autistic individuals were recruited mainly through the online participant platform, Prolific (https://prolific.co). In addition, some snowball sampling was used, as participants were asked to send the study to people who they thought may like to take part.

#### Procedure

The study was presented online using Gorilla, the online platform for behavioural experiments and surveys (https://gorilla.sc/). Participants were given unique log-in details to access an online set of tasks and questionnaires that they could complete at a time and place of their choosing. After providing informed written consent to participate, individuals were presented with a sequence of questionnaires and tasks, including the CQ. As noted above, the language tests were included for a companion analysis (Wilson & Bishop, 2022). There were three language tests devised to target different aspects of receptive language skills, including vocabulary knowledge, grammatical sensitivity and pragmatic understanding of implied meaning.

#### Measures

##### Conversational questionnaire (CQ)

This questionnaire provides the following instructions: “*You will see some statements about people's experiences with conversation*.

- Please choose **MOST SITUATIONS** if the statement applies to most conversations you have with most people.
- Please choose **SOME SITUATIONS** if the statement applies to fewer than half the conversations you have, and/or only when speaking with some people, e.g., strangers.
- Please choose **RARELY / NEVER** if the statement does not apply to you much.

*Please try and fill in all the questions. At the bottom of each page, you can give any comments. Don't spend too long on any one statement. Just give your first impression.*” Most items target social/pragmatic aspects of conversation (46 items; please see Appendix 1 for the full questionnaire). Alongside these items, there are two subscales of control items that we might not expect individuals with social/pragmatic difficulties to necessarily endorse. These control sub-scales were intended to measure Core Language Difficulties (speech and grammar; example item: *“I leave off parts of words, even when I am not stressed. I might say “dent” instead of “accident”.”*) and Negative Interaction Style (being deliberately oppositional or hurtful in one’s communication; example item: *“I spread rumours about people.”*). Responses for items are converted to scores of 0 (RARELY/NEVER), 1 (SOME SITUATIONS) or 2 (MOST SITUATIONS). See Appendix 1 for the version of the questionnaire given to participants in this study.

#### Further questionnaire measures

##### Autism spectrum quotient-10 (AQ-10; Allison et al., 2012)

This 10-item questionnaire measures autistic traits. In the original validation study, the measure had 85% correct discrimination between almost 450 autistic adults and over 800 control adults. The National Institute for Health and Care Excellence (2012) recommend use of the questionnaire for identifying individuals for comprehensive autism assessment. A clinical cut-off of 6 or more is taken as indicating possible autism.

##### Communication checklist - self report (CC-SR; Bishop et al., 2009)

This is a norm-referenced questionnaire measuring self-reported communication challenges. In this study, participants were only presented with the pragmatic language scale (22 items). For each item, participants identify how frequently certain communication behaviours apply to them on a 4-point scale from “less than once a week (or never)” to “several times a day (or all the time)”. An example item is *“People tell me that I ask the same question over and over”*. Total scores are converted to z-scores based on the standardisation sample.

##### Adult reading questionnaire (ARQ) reading scale (*Snowling* et al., 2012)

This 5-item questionnaire measures self-reported reading difficulties. In the original validation study, it showed good construct validity (correlating with observed literacy ability at − 0.67) and, along with self-reported dyslexia status, discriminated with 88% accuracy in identifying those with weaker literacy skills. In the current study, a score of 6 was taken to indicate reading difficulties; this translates to over 1.5 SDs above the mean in individuals not self-reporting dyslexia in the original validation study.

##### Adult ADHD self-report screening scale for Diagnostic and Statistical Manual of Mental Disorders (DSM)-5 (ASRS-5; Ustun et al., 2017)

In this 6-item questionnaire, participants indicate how frequently they experience certain characteristics of attention deficit hyperactivity disorder (ADHD). These are rated on a 5-point scale from “never” to “very often”. An example item is “*How often do you put things off to the last minute?*” In a community sample of over 300 individuals, the measure showed sensitivity and specificity of over 90% to ADHD (with somewhat lower specificity when used in a clinical sample). A cut-off of 14 indicates possible ADHD.

##### Generalised anxiety disorder-7 (generalised anxiety disorder (GAD)-7; Spitzer et al., 2006)

In this 7-item questionnaire, participants rate how frequently they have experienced symptoms of anxiety in the past two weeks. Individuals give ratings on a 4-point scale from “not at all” to “nearly every day”. An example item is “*feeling nervous, anxious or on edge*”. In a primary care sample of over 900 people, a cut-off of 10 gave sensitivity of almost 90% and specificity of over 80% for generalised anxiety disorder (GAD).

##### Short version of the social phobia inventory (Mini-SPIN; Connor et al., 2001)

In this 3-item questionnaire, participants rate how frequently they have experienced symptoms of social anxiety in the past week. Individuals give ratings on a 5-point scale from “not at all” to “extremely”. An example item is “*I avoid activities in which I am the center of attention*”. In a sample of over 1000 managed care patients, the scale gave 90% accuracy in distinguishing individuals with and without social anxiety disorder. A cut-off of 6 indicates possible social anxiety disorder.

##### Intolerance of uncertainty scale (IUS-12; Carleton et al., 2007)

In this self-report measure of intolerance of uncertainty, participants are presented with 12 statements about uncertainty, ambiguous situations, and the future. They rate how closely each statement relates to them on a 5-point scale from “not at all characteristic of me” to “entirely characteristic of me”. An example item is: “*When I am uncertain, I can’t function very well.*”

#### Cognitive/language tests

##### International cognitive ability resource (ICAR) sample test (Condon & Revelle, 2014)

This is an open-access test of general cognitive ability, comprising 16 items. The test is in a multiple-choice format and includes 4-item subtests of four item types: matrix reasoning, verbal reasoning, three-dimensional rotation, and letter-number sequences. Participants score one point for each correct answer. In a large online sample, the ICAR Sample Test had good internal consistency (alpha = 0.81), and good convergent validity (correlating at approximately 0.8 with a commercial IQ measure when correcting for reliability and restriction of range). As young college students were significantly over-represented in the validation study, population norms cannot be adequately generated from the dataset, but summary statistics derived from that sample (*M* = 8.21, *SD* = 3.77) offer a useful point of comparison for the present study.

##### Synonyms test (Wilson & Bishop, 2019)

This is a 25-item test of vocabulary knowledge used to measure receptive vocabulary knowledge. Participants select which of five written words is synonymous with a target word, under a 12 s time limit. Participants score one point for each correct response. The original version of the Grammaticality Decision Test (described below) and this task showed a moderate correlation in both autistic and non-autistic samples, suggesting they are overlapping measures of core language ability (Wilson & Bishop, 2019, 2021).

##### Implicature comprehension test-2 (ICT-2; Wilson & Bishop, 2022)

In this test of pragmatic language comprehension, participants are asked to interpret implied meaning in short conversational adjacency pairs. In the 40 items, the first character asks a closed question (eliciting a “yes” or “no” answer) and the second character produces a short answer without directly saying “yes” and “no”. Following the dialogue, the participant hears a comprehension question to test whether they understood the implied meaning. They answer using a 4-point scale (“yes”, “maybe yes”, “maybe no”, “no”) by clicking buttons arranged horizontally on the screen. Example:

Character 1: Did you hear what the police said?

Character 2: There were lots of trains going past.

Comprehension Question: Did he hear what the police said?

Answer: No

Half the comprehension questions are correctly answered by “yes” and half by “no”. There are two measured variables: total accuracy and total confidence. For total accuracy, participants’ responses are collapsed according to polarity, such that both “yes” and “maybe yes” are counted as accurate if an item is correctly answered by “yes”, and vice versa for “no”. Participants score 1 point for each accurate response (for a total out of 40). For total confidence, participants score 1 point for each “yes” and “no” response, regardless of polarity (for a total out of 40).

##### Grammaticality decision test (GDT; Wilson & Bishop, 2022)

In this test of core language ability, participants listen to a sequence of 50 sentences and decide if the sentence is grammatical or not. Half the sentences are grammatical. Grammatical violations represent mistakes that native speakers would not tend to make, such as using an incorrect verb form (e.g., I went out after I have eaten dinner) or atypical placing of adverbs (e.g., If you can’t find it, I can send again the letter). Participants are asked whether the sentences are grammatical, indicating “yes”, “maybe yes”, “maybe no” and “no” as their answer by clicking buttons arranged horizontally on the screen, as in the ICT-2. Total accuracy and total confidence are computed for this test in a similar way to the ICT-2 (for totals out of 50).

For a summary of all measures presented alongside the CQ and their associated construct, see Table 2.

**Table 2.** Measures presented in the survey alongside the Conversation Questionnaire (CQ).

Test	Variable	Construct
Autism Spectrum Quotient-10 (Allison et al., 2012)	AQ-10 Total	Self-reported autistic traits
Communication Checklist - Self Report (Bishop et al., 2009)	CC-SR Pragmatic Z-score	Self-reported difficulties with pragmatics/communication
Adult Reading Questionnaire (Snowling et al., 2012)	ARQ Reading Scale Total	Self-reported reading difficulties
Adult ADHD Self-Report Screening Scale for DSM-5 (Ustun et al., 2017)	ASRS-5 Total	Self-reported ADHD traits
Generalised Anxiety Disorder-7 (Spitzer et al., 2006)	GAD-7 Total	Current symptoms of generalised anxiety
Short version of the Social Phobia Inventory (Connor et al., 2001)	Mini-SPIN Total	Current symptoms of social anxiety
Intolerance of Uncertainty Scale (Carleton et al., 2007)	IUS-12 Total	Trait-level differences in comfort/discomfort with uncertainty
International Cognitive Ability Resource Sample Test (Condon & Revelle, 2014)	ICAR Total	General cognitive ability
Synonyms Test (citations included in-text)	Synonyms Test Total	Vocabulary knowledge/verbal ability
Implicature Comprehension Test-2 (removed for peer review)	1) ICT-2 Total Accuracy 2) ICT-2 Total Confidence	Pragmatic language comprehension (inferring implied meaning)
Grammaticality Decision Test (removed for peer review)	1) GDT Total Accuracy 2) GDT Total Confidence	Core language ability (sensitivity to grammatical norms)

*Note.* AQ-10* *=* *Autism-Spectrum Quotient-10; CC-SR* *=* *Communication Checklist – Self Report; ARQ* *=* *Adult Reading Questionnaire; ASRS-5* *=* *Adult ADHD Self-Report Screening Scale for DSM-5; GAD-7* *=* *Generalised Anxiety Disorder-7; Mini-SPIN* *=* *Short version of the Social Phobia Inventory; IUS-12* *=* *Intolerance of Uncertainty Scale; ICAR* *=* *International Cognitive Ability Resource; ICT-2* *=* *Implicature Comprehension Test-2; GDT* *=* *Grammaticality Decision Test.

#### Data analysis

Data and the analysis script can be found on the Open Science Framework: https://osf.io/uqyt9/. Analysis was completed in the statistic environment R (R Core Team, 2021).

The first analysis step involved assessing the psychometric structure and reliability of the CQ social/pragmatic items, based on the item response theory approach (IRT; Embretson & Reise, 2000). R package mirt was used for this purpose (Chalmers, 2012). As the data are ordinal, a graded response model was used to model the 46 social/pragmatic items. The number of dimensions present in the data was assessed, first by inspecting a scree plot of eigenvalues, and then comparing fit statistics for models with one, two, three and four dimensions. In terms of fit statistics, corrected Akaike Information Criterion (AIC) and Bayesian Information Criterion (BIC) were computed by function mirt() (Chalmers, 2012). As described below, a graded response model with two dimensions was preferred. Loadings (discrimination parameters) were reviewed for each social/pragmatic item to identify items that did not fit well (i.e., had loadings less than 0.5) and items with lower loadings were dropped from the final version of the measure. The fit statistics Comparative Fit Index (CFI) and Root Mean Square Error of Approximation (RMSEA) were determined for the overall model using the M2 function, and reliability was estimated using the empirical_rxx function for each dimension (Chalmers, 2012). As the final model included two dimensions, it was useful to ask whether a single total score could summarise performance across the social/pragmatic items. This was assessed by comparing Cronbach’s alpha and Revelle’s beta for the items, computed using R package psych (Revelle, 2020). Cronbach’s alpha is calculated based on the average inter-item correlation but can give an inflated impression of test consistency if there are “testlets” (groups of items that are more highly correlated with each other than to other items), whereas Revelle’s beta is an estimate of the worst split-half reliability and gives an indication of general factor saturation in a test (Revelle, 1979). Therefore, if there is high discrepancy between alpha and beta, we can infer that a total score is unlikely to be representative of the test, whereas similar values would support use of a total score.

After completing these initial analyses of the structure of the questionnaire, validity was considered. This involved (1) assessing how well the CQ dimensions discriminated between groups where a difference might be expected, and (2) looking at the relationship between CQ dimensions and other measures included in the study. In terms of (1), the aim was to assess how well the questionnaire discriminated between groups where a difference might be expected (i.e., between autistic and non-autistic people). This involved computing Cohen’s *d* and sensitivity and specificity associated with receiver operating characteristic (ROC) curves. ROC analysis was carried out using R package ROCR (Sing et al., 2005). In terms of (2), correlations were assessed between the CQ dimensions and measures we expect to be related (autistic traits measured by the AQ-10 and communication challenges measured by the CC-SR) and measures we expected to be relatively unrelated (self-reported reading difficulties measured by the ARQ Reading Scale, receptive core language skills on the Synonyms Test and GDT, and general cognitive ability on the ICAR).

## Results

Data were analysed where participants had provided responses to at least 42 of 46 social/pragmatic items of the CQ (i.e., over 90% of the test). This meant that data for 312 participants were retained for the analysis, with eight excluded.

### Analysis of dimensions/themes in the CQ and identification of items for final inclusion in the CQ

The first stage of analysis considered the number of dimensions present across the social/pragmatic items. This involved carrying out eigenvalue decomposition of the inter-item correlations. There was one very large eigenvalue (19.00), a second eigenvalue substantially over one (3.54), and three other eigenvalues over one (1.51, 1.24, and 1.09). This pattern of eigenvalues suggests that a general factor is likely to account well for responses on the questionnaire, but there may be at least one additional group factor. Graded response models with differing numbers of dimensions were used to identify the model showing the best fit. As shown in Table 3, a two-dimensional model seemed most appropriate; it was parsimonious while showing good fit.

**Table 3.** Fit statistics for graded response models of the social/pragmatic items of the CQ.

Number of Dimensions	Corrected AIC	BIC
One	23,225.07	23,519.85
Two	22,644.94	22,803.78
Three	23,231.28	22,848.66
Four	25,464.40	22,905.74

*Note.* Models were tested where there are either one or multiple dimensions accounting for response patterns across the social/pragmatic items of the CQ. Smaller values indicate better fit. AIC = Aikake Information Criterion, BIC = Bayesian Information Criterion.

See Table 4 for item loadings for this two-dimensional model.

**Table 4.** Factor loadings for each social/pragmatic item of the CQ.

Item	Statement	CQ Social/Pragmatic Dimension One	CQ Social/Pragmatic Dimension Two
**1**	**I get confused when people give hints or say things indirectly.**	0.40	**0.51**
2	I get lost when the topic of conversation changes.	0.39	0.48
3	I don’t understand jokes or sayings.	0.29	0.43
**4**	**I find it hard to speak at length, so only say one or two things at a time.**	**0.81**	−0.21
**5**	**It takes me a long time to decide what to say next in a conversation.**	**0.79**	0.01
**6**	**I don’t know how to start conversations with people.**	**0.85**	−0.02
**7**	**If someone interrupts me when I’m talking, I have to start at the beginning again.**	0.21	**0.59**
8	I have much more difficulty than other people my age remembering words I need in conversations.	0.29	0.42
**9**	**In conversations, I like there to be a purpose. I find it hard when it’s just “social”.**	**0.62**	0.29
10	When I chat to others, I feel I am playing a role that is not me.	0.47	0.27
**11**	**I talk about random or unrelated topics. People find it difficult to follow.**	0.09	**0.73**
**12**	**I analyse what other people mean, because I don’t understand or think I’ve misunderstood.**	0.30	**0.61**
**13**	**I feel unsure whether I have got my point across correctly.**	0.39	**0.51**
14	I don’t know what to say when someone tells me how they feel.	0.44	0.31
**15**	**I take things literally.**	0.23	**0.58**
**16**	**I find conversation tiring. I feel like I need time to recover afterwards.**	**0.57**	0.29
17	I think most things people say are not relevant to me.	0.45	0.42
**18**	**I get into confrontations without meaning to.**	−0.01	**0.75**
**19**	**I talk in much longer stretches than other people do.**	−0.35	**0.93**
**20**	**I have to say exactly what I think, even if I might get into trouble.**	−0.16	**0.72**
21	I get mixed up when forming my thoughts into sentences.	0.45	0.38
**22**	**I find it hard to think of good questions to keep a conversation going.**	**0.84**	0.06
**23**	**I struggle to think of a polite way to say things. I might come across as blunt and rude.**	0.20	**0.63**
**24**	**During conversations, I lose track of what other people know or might be thinking.**	0.38	**0.61**
**25**	**I get frustrated when people don’t answer my questions properly.**	0.15	**0.63**
**26**	**If people say things that don’t match their body language or behaviour, I get really confused.**	0.28	**0.61**
**27**	**I don’t know what to say in groups.**	**0.83**	0.04
28	I find it hard to change how I speak for different people.	0.35	0.47
**29**	**I find it hard to join a conversation. I might interrupt or say nothing.**	**0.72**	0.18
**30**	**I say things out of context and people are not sure what I mean.**	0.22	**0.71**
**31**	**I give lots more detail than other people do.**	−0.07	**0.82**
**32**	**I find it hard to talk about my feelings.**	**0.57**	0.07
**33**	**I talk too much and the other person doesn’t get a turn.**	−0.28	**0.90**
34	I forget ways to vary a conversation. For instance, I may forget to ask questions.	0.43	0.49
**35**	**My point comes out wrongly when I respond to someone quickly.**	0.35	**0.59**
36	It takes me a long time to process what people are saying.	0.47	0.45
**37**	**I can’t think of comments or experiences to tell people in conversation.**	**0.92**	−0.10
**38**	**Unless I really need to, I prefer not to talk.**	**0.77**	−0.19
**39**	**I sometimes cut in or speak over people when I don’t mean to.**	−0.12	**0.74**
**40**	**I have no interest in everyday chat, e.g., about the weekend.**	**0.59**	0.25
**41**	**It is hard finding common ground when talking to people.**	**0.65**	0.28
**42**	**I struggle to think of things to say on the spot.**	**0.93**	−0.01
**43**	**I lose track of what I am saying.**	0.22	**0.52**
**44**	**There are particular things I like to talk about, but people are rarely interested.**	0.21	**0.64**
**45**	**I can’t judge what topics are appropriate to talk about.**	0.26	**0.69**
**46**	**When I have something I want to say, I can’t find an opportunity to say it.**	**0.57**	0.34

Note. All the social/pragmatic items of the CQ have been modelled in a two-dimensional graded response model. Items in bold type are retained in the final version of the CQ. Bold type is used to indicate the dimension to which each item is taken to belong.

Ten items showed only modest loadings on the dimensions (less than 0.5), and often cross-loaded, so these were excluded from the final analysis. This left 36 items, with 15 loading more strongly on Social/Pragmatic Dimension One, and 21 on Social/Pragmatic Dimension Two. The two dimensions were reviewed by the researcher to identify what the common themes seemed to be across the items. Social/Pragmatic Dimension One seemed to represent difficulties knowing what to say in conversation, whereas the second dimension seemed to reflect misunderstanding what someone says or using behaviours that may impact the conversation (e.g., being blunt, talking in longer stretches than others or speaking on random/unrelated topics). A final graded response model was run including just the 36 social/pragmatic items retained in the final questionnaire. Fit statistics for this model were excellent, CFI = 0.98, RMSEA [90% CI] = 0.055 [0.049, 0.060]. Factor scores were extracted for the two dimensions from the model, and showed a strong correlation, *r* = 0.65, *p* < .001. (It would also be fine to compute raw totals for the two dimensions just by adding scores for items associated with that dimension rather extracting factor scores from the IRT model; correlations between the factor score and raw total were near perfect for each dimension, *r* = 0.99, *p* < .001.)

### Analysis of reliability of the CQ

The social/pragmatic items showed high internal reliability. IRT reliability coefficients for Social/Pragmatic Dimensions One and Two were both 0.93. When considering all social/pragmatic items together rather than in separate dimensions, reliability coefficients were also high. Cronbach’s alpha was 0.96 and Revelle’s beta was 0.89. As these indices are similar to each other, it suggests that the CQ items show high internal consistency and high general factor saturation, suggesting that one total score can appropriately summarise a person’s response pattern on the questionnaire if clinicians/researchers preferred to use just one value.

### Analysis of control items in the CQ

Next, attention shifted to the control items to see how well these functioned in the questionnaire. Raw totals were computed for the Core Language Difficulties and Negative Interaction Style sub-scales (four items each, to give totals out of eight). As a lower score on the Social/Pragmatic Dimensions indicated greater challenges, totals on the control sub-scales were multiplied by minus one to produce negative values so that lower scores would also indicate greater challenges on these sub-scales. Correlations between the Social/Pragmatic Dimensions and the subscales for Core Language Difficulties and Negative Interaction Style were modest, as shown in Table 5. In addition, relatively few people endorsed these items, as expected. For core language, 62.5% scored 0 on this scale, and only 5.4% scored over 4 (out of 8). For negative interaction style, 75% scored 0 on this scale, and nobody scored over 4 (out of 8).

**Table 5.** Correlations between the two control subscales of the CQ and the Social/Pragmatic Dimensions of the CQ.

	Social/Pragmatic Dimension One	Social/Pragmatic Dimension Two
CQ Core Language Difficulties	0.30 ***	0.41 ***
CQ Negative Interaction Style	0.08	0.26 ***

*Note.* * = *p* < 0.05; ** = *p* < 0.01; ** = *p* < 0.001

### Comparison of group scores on the CQ

The next step of analysis focused on how the different groups performed on the CQ. First, see Table 6 for descriptive statistics characterising the groups on the range of measures administered in this study.

**Table 6.** Descriptive statistics for each group.

	Group 1, Autistic (*N* = 101)		Group 2, Self-diagnosed autistic (*N* = 34)		Group 3, Reading Difficulties (*N* = 49)		Group 4, Control (*N* = 110)		Group 5, Non-autistic high AQ (*N* = 18)	
	Mean	*SD*	Mean	*SD*	Mean	*SD*	Mean	*SD*	Mean	*SD*
Age (years)	41.69	14.28	41.94	13.31	34.61	15.51	38.11	14.45	39.67	13.58
AQ-10 Total	7.73	2.15	6.85	2.56	3.20	1.31	2.31	1.44	6.83	1.20
CC-SR Pragmatic Z-score ^a^	-2.36	1.73	-2.14	1.65	-0.64	1.08	0.01	1.12	-0.91	1.05
ARQ Reading Scale Total	4.34	3.13	4.18	3.48	7.84	2.10	2.28	1.49	5.50	3.43
ASRS-5 Total	14.41	5.06	13.91	4.34	10.92	4.24	8.60	3.72	10.78	3.41
GAD-7 Total ^a^	10.35	6.06	8.56	5.94	7.25	5.21	5.10	4.22	8.72	7.06
Mini-SPIN Total ^a^	5.76	3.76	6.09	3.22	5.15	3.22	3.22	2.98	5.28	4.34
IUS-12 Total	33.29	9.32	30.89	7.17	23.93	7.69	22.08	9.43	28.60	6.14
ICAR Total	8.95	3.89	9.09	3.96	6.53	3.68	8.46	3.43	8.56	3.47
Synonyms Test Total	15.72	5.12	15.50	5.76	10.92	5.11	13.38	4.73	13.67	5.06
ICT-2 Total Accuracy	35.60	4.29	35.47	3.43	36.96	1.97	37.33	2.22	36.17	2.46
ICT-2 Total Confidence	18.53	9.83	19.44	9.35	24.49	10.66	23.51	9.41	22.28	7.04
GDT Total Accuracy	43.48	4.81	42.50	6.55	40.98	4.60	43.20	5.25	40.72	6.82
GDT Total Confidence	45.77	6.18	45.12	7.77	45.18	5.21	46.74	3.89	43.67	7.18
CQ Social/Pragmatic Dim. One	-0.89	1.16	-0.54	1.05	0.25	1.28	0.97	1.19	-0.07	1.09
CQ Social/Pragmatic Dim. Two	-1.02	1.14	-0.70	1.19	0.25	1.13	1.14	0.95	-0.28	1.10
CQ Core Language Difficulties	-1.27	2.05	-1.09	1.69	-1.48	1.77	-0.40	1.08	-1.17	1.10
CQ Negative Interaction Style ^a^	-0.46	0.90	-0.21	0.48	-0.42	0.85	-0.22	0.57	-0.56	0.78

*Note.* Higher scores on the AQ-10, ARQ, ASRS-5, GAD-7, Mini-SPIN, and IUS-12 (but lower scores on the CC-SR) indicate higher levels of the particular feature. Higher scores on the ICAR, Synonyms Test, ICT-2 and GDT indicate stronger performance on these cognitive/language measures. For the CQ, factor scores are presented. These were extracted from the final version of the graded response model; lower scores indicate more self-reported challenges with that aspect of conversation.

^a^ Sample sizes were slightly smaller for these variables due to missing data. *N*s for each variable were: CC-SR Pragmatic Z-score = [97, 32, 47, 107, 15]; GAD-7 Total = [97, 32, 48, 109, 18]; Mini SPIN Total = [99, 34, 48, 109, 18]; CQ Core Language Difficulties = [101, 34, 48, 108, 18]; CQ Negative Interaction Style = [99, 34, 48, 107, 18].

**Figure 1.**
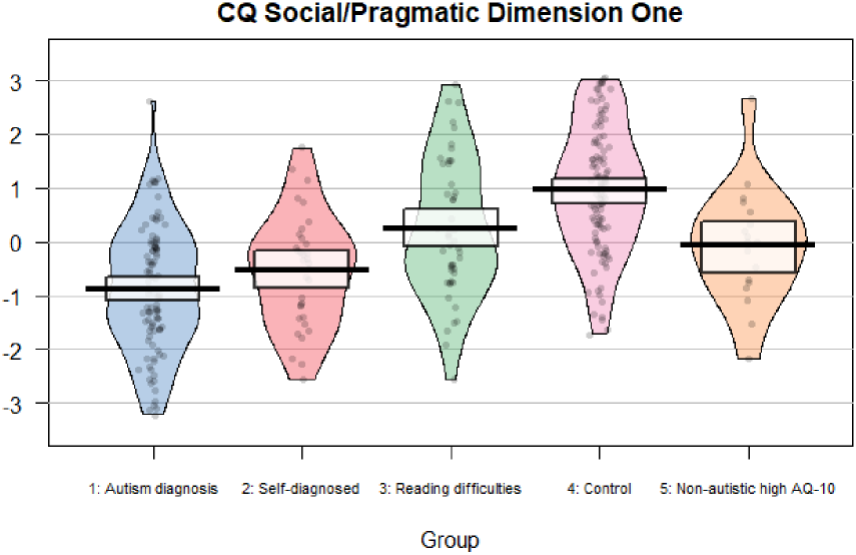
Plot showing factor scores for Social/Pragmatic Dimension One of the CQ for all participants by group.

**Figure 2.**
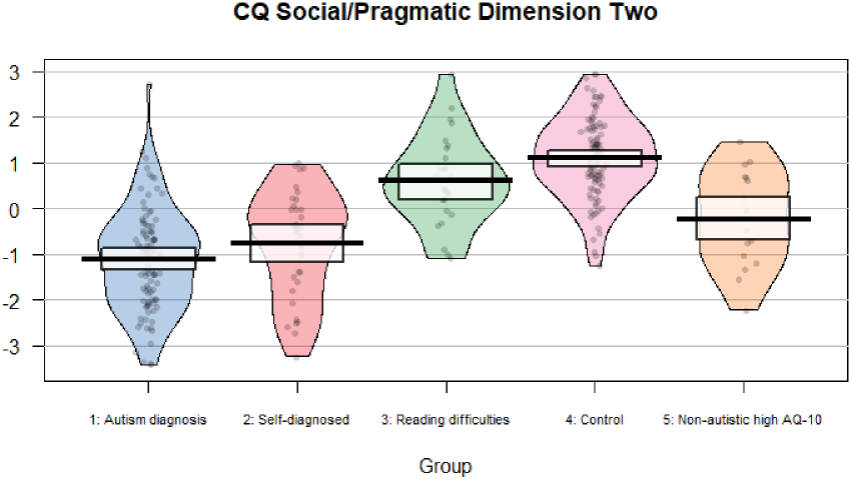
Plot showing factor scores for Social/Pragmatic Dimension Two of the CQ for all participants by group.

See Figures 1 and 2 for pirate plots showing data for each participant on the Social/Pragmatic Dimensions of the CQ. There were large effect size differences between the group with autism diagnoses and the control group for Social/Pragmatic Dimension One, *t* (208.37) = 11.50, *p* < .001, *d* = 1.59, and Social/Pragmatic Dimension Two, *t* (195.28) = 14.84, *p* < .001, *d* = 2.07. ROC analysis indicated that questionnaire responses could distinguish effectively between these groups (Social/Pragmatic Dimensions One, Area Under the Curve (AUC) = 0.87, and Two, AUC = 0.93), with optimal cut-offs of 16 and 18 for the two dimensions. The CQ was less effective in distinguishing the group with autism diagnoses and the group with self-reported reading difficulties (Social/Pragmatic Dimensions One, AUC = 0.77, and Two, AUC = 0.79), with slightly higher optimal cut-offs of 18 and 20. See Table 7 for sensitivities and specificities for different cut-offs on the two dimensions.

**Table 7.** Sensitivities and specificities of the CQ dimensions at different cut-off*s*.

Dimension One				Dimension Two			
Cut-off	Sensitivity (%)	neurotypical control group (Group 4) (%)	Specificity compared to group with reading difficulties (Group 3) (%)	Cut-off	Sensitivity (%)	neurotypical control group (Group 4) (%)	Specificity compared to group with reading difficulties (Group 3) (%)
14	80	73	47	16	87	85	53
15	77	78	51	17	84	88	58
16	74	83	58	18	83	92	60
17	68	85	60	19	81	92	67
18	63	88	79	20	80	93	69
19	58	90	79	21	72	95	71
20	53	92	88	22	71	96	73

### Analysis of convergent and discriminant validity

Finally, convergent and discriminant validity of the CQ was assessed through correlation analysis. See Table 8 for values. As expected, both Social/Pragmatic Dimensions showed a strong relationship with scores on the AQ-10 (a measure of autistic traits) and the pragmatic scale of the CC-SR (a measure of communication difficulties), all *r*s > 0.50. Likewise, both dimensions show low relationships with the GDT and Synonyms Test (measures of receptive core language skills) and the ICAR (a measure of general cognitive ability), all *r*s < 0.20.

**Table 8.** Correlations between CQ Social/Pragmatic Dimensions and other variables*.*

	CQ Social/Pragmatic Dimension One	CQ Social/Pragmatic Dimension Two
AQ-10 Total	-0.59 ***	-0.67 ***
CC-SR Pragmatic Z-score	0.47 ***	0.73 ***
ARQ Reading Scale Total	-0.30 ***	-0.30 ***
IUS-12 Total	-0.55 ***	-0.55 ***
ASRS-5 Total	-0.46 ***	-0.63 ***
GAD-7 Total	-0.39 ***	-0.50 ***
Mini-SPIN Total	-0.48 ***	-0.38 ***
ICAR Total	-0.04	0.07
Synonyms Test Total	-0.08	-0.01
ICT-2 Total Accuracy	0.25 ***	0.32 ***
ICT-2 Total Confidence	0.26 ***	0.29 ***
GDT Total Accuracy	0.01	0.08
GDT Total Confidence	0.17 **	0.16 **

*Note.* * = *p* < 0.05; ** = *p* < 0.01; ** = *p* < 0.001.

## Discussion

In this study, a new Conversation Questionnaire (CQ) was developed in a collaborative effort with autistic people, and there was some initial validation of the questionnaire. Autistic people helped develop questionnaire items relevant to the challenges they experience in conversation. They provided feedback on the questionnaire, indicating they felt it was acceptable and sensitively written, and showed good face validity (i.e., it seemed to represent the range of their difficulties well). Analysis in *Part II* of the study showed that these challenges fell into two broad dimensions: (1) difficulties finding things to say in conversation and (2) engaging in behaviours that may be disruptive to neurotypical conversation (e.g., being blunt, over-dominant in conversation). This tells us something new about the nature of communication skills, as there is limited research into the factor structure of communication behaviours. An exception to this is research using the Broad Autism Phenotype Questionnaire (BAPQ). With this questionnaire, it has been possible to measure a preference to engage in less social communication (called Aloof Behaviour in the BAPQ) as a distinguishable phenomenon from behaviour that may be seen as more overtly “disruptive” to neurotypical interactions (Pragmatic Language Problems in the BAPQ; Hurley et al., 2007; Sasson et al., 2013). The CQ makes a similar distinction.

The CQ shows good psychometric properties. The two-dimensional structure fitted the data well, and showed excellent internal reliability in a large sample (over 300 people). The questionnaire also showed promising evidence of validity. Scores were closely related to variables that we would expect, including self-reported autistic traits and another measure of self-reported communication difficulties (CC-SR pragmatic scale). In addition, scores seemed to be specific to conversational challenges and did not pick up difficulties with aspects of formal language ability (vocabulary and grammar skills) or general cognitive ability, and were only weakly associated with self-reported reading skills. This suggests good convergent and discriminant validity. In addition, the CQ dimensions were very good at distinguishing between those with a clinical diagnosis of autism and the neurotypical control group. Autistic people scored more highly on both the dimensions in the present study with large effect sizes, and scores on these dimensions were associated with good sensitivity and specificity for autism when compared to neurotypical people in our sample. However, the CQ should not be viewed as a comprehensive diagnostic measure for autism as it does not tap the full autism phenotype (i.e., it does not address restricted and repetitive behaviours and interests) and will be limited by the biases associated with self-report that would be relevant to any population. It would need to be integrated into a multi-method assessment for autism.

In addition to individuals with a clinical diagnosis of autism, this study also included people self-diagnosed with autism, those with elevated autistic traits and individuals with self-reported reading difficulties/dyslexia. All these groups scored higher on the CQ than the neurotypical group, suggesting that the questionnaire picks up conversation challenges that are to some extent shared across different neurodiverse presentations. This agrees with the view that different aspects of neurodiversity are overlapping rather than distinct (Thapar et al., 2017). It was possible that the dyslexic group would not show elevated scores on the CQ, as the core features of dyslexia relate to literacy rather than social communication. However, pragmatic difficulties have previously been found in dyslexia in several small studies (Cappelli et al., 2018; Cardillo et al., 2018; Griffiths, 2007), and this study supports the view that dyslexic individuals are likely to have broader language and communication needs than just literacy-related. Overall, it seems the CQ may have utility in assessing communication skills, needs and preferences across heterogeneous groups of neurodiverse people. It is worth noting that communication itself is heterogeneous, and a further strength of the CQ may be its inclusion of Core Language and Negative Interaction Style sub-scales alongside the Social/Pragmatic Dimensions. These sub-scales were originally included to flag cases where items might have been endorsed indiscriminately (perhaps due to poor attention, misunderstanding or a reporting bias). However, these sub-scales may have further use in terms of providing greater insight into the specifics of an individual’s communication needs – for instance, if the person experiences issues with grammar/speech or tends to interact in an oppositional way, alongside experiencing the core pragmatic differences in autism.

### Uses of the conversation questionnaire (CQ)

- The CQ may support in diagnosing autism as part of a holistic assessment, alongside observational and interview measures, including assessment of other core aspects of autism such as repetitive and restricted behaviours and interests. Unlike other tools, the CQ has been co-produced with autistic people, so it may offer a novel perspective.
- The CQ may help identify strengths and difficulties in communication as part of a speech and language assessment with a range of neurodiverse people.
- The CQ may offer a therapeutic tool enabling clients to reflect on their communication skills, perhaps to identify adjustments/adaptations they may need in day-to-day life and/or identify targets for speech and language therapy.
- The CQ could be used in research relating to language, pragmatics and communication.
- Clinicians should be cautious about using the CQ with adults who struggle with core aspects of language (e.g., grammar, vocabulary, speech), as these individuals were under-represented when devising and validating the questionnaire. The CQ may be appropriate for these individuals if adapted, but this would need to be tested through research. The CQ should not be used to assess core aspects of language.

### Limitations

As this study is simply an initial psychometric evaluation of the questionnaire, it will be important to replicate and extend the results to establish the utility of the questionnaire. Future research might involve testing how well the two-dimensional structure of the questionnaire replicates in other samples; how consistent scores are across repeated administrations; and how different clinical groups perform on the questionnaire. There are also two possible issues with representativeness in this study that might be important to consider further, as the questionnaire was developed and validated with convenience samples skewed towards educated white females. Historically, it has been suggested that there are sex differences in language and social communication abilities, so we might question whether the over-representation of women affects the representativeness of the data. However, it should be noted that empirical research has generally not supported the idea of sex differences in verbal abilities in the general population (Wallentin, 2009) and evidence for sex differences in social communication difficulties remains limited and inconclusive, and such evidence seems to depend on the measures used (e.g., Hull et al., 2017a; Mahendiran et al., 2019; Wood-Downie et al., 2021). It is therefore difficult to conclude how significant the skewed gender distribution of the sample is.

The limited diversity in terms of culture, race and ethnicity, and level of education/cognitive ability may also have impacted on the study. For instance, social norms have a significant influence on social communication, so autistic individuals from different cultural/ethnic backgrounds may report different challenges, which are not represented in the CQ due to the make-up of the sample. It is worth noting that the CQ does include open free response boxes at the bottom of each page, and people are invited to expand on answers and give detail on any further challenges. Participants did use these boxes to give personal examples, but there was no evidence during the validation phase that the CQ was consistently missing certain types of difficulties. Overall, it is not clear whether the skewed nature of the development and validation samples had a meaningful impact in this study, but ideally future research would aim to collect norms in a more representative sample.

The CQ may be less appropriate for autistic people with learning difficulties/disabilities, as they may have challenges with a range of language skills and not just the social/pragmatic aspects of conversation measured by the CQ. They may also find the level of literacy required to complete the CQ challenging. The CQ may need adaptation for individuals with learning/language difficulties or it simply may not measure the experiences and difficulties of these groups in the most relevant way; further research is needed to clarify these issues. In addition to the literacy level of the CQ, the length may also be challenging for some individuals. In this respect, it may be helpful to develop a short version of the CQ including a smaller number of items with the highest loadings on the two dimensions.

In summary, the CQ may be useful to clinicians and researchers measuring communication challenges relevant to autism. The CQ has the advantage of being developed with the insights of autistic people and attuned to the difficulties they experience. As with all self-report questionnaires, this measure would not be appropriate as a diagnostic tool, but it may contribute to an autism assessment. Speech and language therapists (and other clinicians) may also find it a helpful tool in supporting individuals to understand their strengths and difficulties in conversation.

## Supplemental Material

sj-docx-1-dli-10.1177_23969415221123286 - Supplemental material for Development and validation of the conversation questionnaire: A psychometric measure of communication challenges generated from the self-reports of autistic people Click here for additional data file. Supplemental material, sj-docx-1-dli-10.1177_23969415221123286 for Development and validation of the conversation questionnaire: A psychometric measure of communication challenges generated from the self-reports of autistic people by Alexander C Wilson in Autism & Developmental Language Impairments
