# Supplementary material for: Development and validation of the conversation questionnaire: A psychometric measure of communication challenges generated from the self-reports of autistic people
Source: Autism Dev Lang Impair. 2022 Sep 4;7:23969415221123286. doi: 10.1177/23969415221123286 (PMC9685143; doi:10.1177/23969415221123286)
Supplement: sj-docx-1-dli-10.1177_23969415221123286 - Supplemental material for Development and validation of the conversation questionnaire: A psychometric measure of communication challenges generated from the self-reports of autistic people [file sj-docx-1-dli-10.1177_23969415221123286.docx]

**Conversation Questionnaire**

*You will see some statements about people's experiences with conversation.*

- *Please choose* ***MOST SITUATIONS*** *if the statement applies to most conversations you have with most people.*
- *Please choose* ***SOME SITUATIONS*** *if the statement applies to fewer than half the conversations you have, and/or only when speaking with some people, e.g. strangers.*
- *Please choose* ***RARELY / NEVER*** *if the statement does not apply to you much.*

*Please try and fill in all the questions. At the bottom of each page, you can give any comments.*

*Don't spend too long on any one statement. Just give your first impression.*

__________________________________________________________________________________

I get confused when people give hints or say things indirectly.

| MOST SITUATIONS | SOME SITUATIONS | RARELY/NEVER |
| --- | --- | --- |

I get lost when the topic of conversation changes.

| MOST SITUATIONS | SOME SITUATIONS | RARELY/NEVER |
| --- | --- | --- |

I don’t understand jokes or sayings.

| MOST SITUATIONS | SOME SITUATIONS | RARELY/NEVER |
| --- | --- | --- |

I find it hard to speak at length, so only say one or two things at a time.

| MOST SITUATIONS | SOME SITUATIONS | RARELY/NEVER |
| --- | --- | --- |

It takes me a long time to decide what to say next in conversation.

| MOST SITUATIONS | SOME SITUATIONS | RARELY/NEVER |
| --- | --- | --- |

COMMENTS

I make mistakes saying long words, even when I speak slowly. I might say “kero” instead of “kangaroo”.

| MOST SITUATIONS | SOME SITUATIONS | RARELY/NEVER |
| --- | --- | --- |

I don’t know how to start conversations with people.

| MOST SITUATIONS | SOME SITUATIONS | RARELY/NEVER |
| --- | --- | --- |

If someone interrupts me when I’m talking, I have to start at the beginning again.

| MOST SITUATIONS | SOME SITUATIONS | RARELY/NEVER |
| --- | --- | --- |

I have much more difficulty than other people my age remembering words I need in conversations.

| MOST SITUATIONS | SOME SITUATIONS | RARELY/NEVER |
| --- | --- | --- |

In conversation, I like there to be a purpose. I find it hard when it’s just “social”.

| MOST SITUATIONS | SOME SITUATIONS | RARELY/NEVER |
| --- | --- | --- |

When I chat to others, I feel I am playing a role that is not me.

| MOST SITUATIONS | SOME SITUATIONS | RARELY/NEVER |
| --- | --- | --- |

I insult people on purpose.

| MOST SITUATIONS | SOME SITUATIONS | RARELY/NEVER |
| --- | --- | --- |

I talk about random or unrelated topics. People find it difficult to follow.

| MOST SITUATIONS | SOME SITUATIONS | RARELY/NEVER |
| --- | --- | --- |

COMMENTS

I analyse what other people mean, because I don’t understand or think I’ve misunderstood.

| MOST SITUATIONS | SOME SITUATIONS | RARELY/NEVER |
| --- | --- | --- |

I feel unsure whether I have got my point across correctly.

| MOST SITUATIONS | SOME SITUATIONS | RARELY/NEVERa |
| --- | --- | --- |

I don’t know what to say when someone tells me how they feel.

| MOST SITUATIONS | SOME SITUATIONS | RARELY/NEVER |
| --- | --- | --- |

I take things literally.

| MOST SITUATIONS | SOME SITUATIONS | RARELY/NEVER |
| --- | --- | --- |

I find conversation tiring. I feel like I need time to recover afterwards.

| MOST SITUATIONS | SOME SITUATIONS | RARELY/NEVER |
| --- | --- | --- |

I leave off parts of words, even when I am not stressed. I might say “dent” instead of accident.

| MOST SITUATIONS | SOME SITUATIONS | RARELY/NEVER |
| --- | --- | --- |

I think most things people say are not relevant to me.

| MOST SITUATIONS | SOME SITUATIONS | RARELY/NEVER |
| --- | --- | --- |

I get into confrontations without meaning to.

| MOST SITUATIONS | SOME SITUATIONS | RARELY/NEVER |
| --- | --- | --- |

COMMENTS

I talk in much longer stretches than other people do.

| MOST SITUATIONS | SOME SITUATIONS | RARELY/NEVER |
| --- | --- | --- |

I spread rumours about people.

| MOST SITUATIONS | SOME SITUATIONS | RARELY/NEVER |
| --- | --- | --- |

I have to say exactly what I think, even if I might get in trouble.

| MOST SITUATIONS | SOME SITUATIONS | RARELY/NEVER |
| --- | --- | --- |

I get mixed up when forming my thoughts into sentences.

| MOST SITUATIONS | SOME SITUATIONS | RARELY/NEVER |
| --- | --- | --- |

I find it hard to think of good questions to keep a conversation going.

| MOST SITUATIONS | SOME SITUATIONS | RARELY/NEVER |
| --- | --- | --- |

I struggle to think of a polite way to say things. I might come across as blunt and rude.

| MOST SITUATIONS | SOME SITUATIONS | RARELY/NEVER |
| --- | --- | --- |

During conversations, I lose track of what other people know or might be thinking.

| MOST SITUATIONS | SOME SITUATIONS | RARELY/NEVER |
| --- | --- | --- |

I get frustrated when people don’t answer my questions properly.

| MOST SITUATIONS | SOME SITUATIONS | RARELY/NEVER |
| --- | --- | --- |

COMMENTS

If people say things that don’t match their body language or behavior, I get really confused.

| MOST SITUATIONS | SOME SITUATIONS | RARELY/NEVER |
| --- | --- | --- |

I don’t know what to say in groups.

| MOST SITUATIONS | SOME SITUATIONS | RARELY/NEVER |
| --- | --- | --- |

I find it hard to change how I speak for different people.

| MOST SITUATIONS | SOME SITUATIONS | RARELY/NEVER |
| --- | --- | --- |

I say embarrassing things because I want to upset people.

| MOST SITUATIONS | SOME SITUATIONS | RARELY/NEVER |
| --- | --- | --- |

I find it hard to join a conversation. I might interrupt or say nothing.

| MOST SITUATIONS | SOME SITUATIONS | RARELY/NEVER |
| --- | --- | --- |

I say things out of context and people are not sure what I mean.

| MOST SITUATIONS | SOME SITUATIONS | RARELY/NEVER |
| --- | --- | --- |

I give lots more detail than other people do.

| MOST SITUATIONS | SOME SITUATIONS | RARELY/NEVER |
| --- | --- | --- |

I find it hard to talk about my feelings.

| MOST SITUATIONS | SOME SITUATIONS | RARELY/NEVER |
| --- | --- | --- |

COMMENTS

I talk too much and the other person doesn’t get a turn.

| MOST SITUATIONS | SOME SITUATIONS | RARELY/NEVER |
| --- | --- | --- |

I forget ways to vary a conversation. For instance, I may forget to ask questions.

| MOST SITUATIONS | SOME SITUATIONS | RARELY/NEVER |
| --- | --- | --- |

I mix up words like “he” and “him”. I might say “him spoke to me”.

| MOST SITUATIONS | SOME SITUATIONS | RARELY/NEVER |
| --- | --- | --- |

My point comes out wrongly when I respond to someone quickly.

| MOST SITUATIONS | SOME SITUATIONS | RARELY/NEVER |
| --- | --- | --- |

It takes me a long time to process what people are saying.

| MOST SITUATIONS | SOME SITUATIONS | RARELY/NEVER |
| --- | --- | --- |

I can’t think of comments or experiences to tell people in conversation.

| MOST SITUATIONS | SOME SITUATIONS | RARELY/NEVER |
| --- | --- | --- |

Unless I really need to, I prefer not to talk.

| MOST SITUATIONS | SOME SITUATIONS | RARELY/NEVER |
| --- | --- | --- |

I sometimes cut in or speak over people when I don’t mean to.

| MOST SITUATIONS | SOME SITUATIONS | RARELY/NEVER |
| --- | --- | --- |

COMMENTS

I have no interest in everyday chat, e.g. about the weekend.

| MOST SITUATIONS | SOME SITUATIONS | RARELY/NEVER |
| --- | --- | --- |

I get word endings mixed up. I might say “I went ran” instead of “I went running”.

| MOST SITUATIONS | SOME SITUATIONS | RARELY/NEVER |
| --- | --- | --- |

It is hard finding common ground when talking to people.

| MOST SITUATIONS | SOME SITUATIONS | RARELY/NEVER |
| --- | --- | --- |

I struggle to think of things to say on the spot.

| MOST SITUATIONS | SOME SITUATIONS | RARELY/NEVER |
| --- | --- | --- |

I lose track of what I am saying.

| MOST SITUATIONS | SOME SITUATIONS | RARELY/NEVER |
| --- | --- | --- |

There are particular things I like to talk about, but people are rarely interested.

| MOST SITUATIONS | SOME SITUATIONS | RARELY/NEVER |
| --- | --- | --- |

I say things in order to scare people.

| MOST SITUATIONS | SOME SITUATIONS | RARELY/NEVER |
| --- | --- | --- |

I can’t judge what topics are appropriate to talk about.

| MOST SITUATIONS | SOME SITUATIONS | RARELY/NEVER |
| --- | --- | --- |

When I have something I want to say, I can’t find an opportunity to say it.

| MOST SITUATIONS | SOME SITUATIONS | RARELY/NEVER |
| --- | --- | --- |

COMMENTS
